# Pregnant Women’s Health Literacy in the South of Iran 

**Published:** 2017-12

**Authors:** Sakineh Dadipoor, Ali Ramezankhani, Azin Alavi, Teamur Aghamolaei, Ali Safari-Moradabadi

**Affiliations:** 1 Mother and Child Welfare Research Center, School of Public Health, Hormozgan University of Medical Sciences, Bandar Abbas, Iran; 2 Department of Public Health, School of Public Health, Shahid Beheshti University of Medical Sciences, Tehran, Iran; 3 Fertility and Infertility Research Center, Faculty of Medicine, Hormozgan University of Medical Sciences, Bandar Abbas, Iran; 4 Social Determinants on Health Promotion Research Center, Faculty of Health, Hormozgan University of Medical Sciences, Bandar Abbas, Iran; 5 Student Research Committee, School of Public Health, Shahid Beheshti University of Medical Sciences, Tehran, Iran

**Keywords:** Health Literacy, Pregnancy, Test of Functional Health Literacy in Adults, Iran

## Abstract

**Objective:** Investigate the health literacy of pregnant women in the south of Iran.

**Materials and methods:** The present descriptive-analytical study was conducted on 775 pregnant women in the south of Iran (including Boushehr, Ahvaz, Zahedan and Bandar Abbas cities) through the clustering sample selection method. The data were collected through the Test of Functional Health Literacy in Adults: TOFHLA, and were analyzed statistically via SPSS 16 through independent-sample t-test and ANOVA.

**Results:** The average age of the subjects was 31.89 ± 7.54 years. The results indicated that 15.5% of the subjects had an inadequate level of health literacy; 41.7% had a border-line level of health literacy, and 42.8% had an adequate level of literacy. Significant correlations were found between each of these variables and the subjects’ level of health literacy: education, age, occupation and care provision during pregnancy (p ˂ 0.05). The highest mean score of health literacy belonged to Ahvaz while the lowest belonged to Boushehr.

**Conclusion:** According to the results, the health literacy level of pregnant women in the target geographical areas showed to be inadequate or on a border-line. Moreover, subjects’ education, age and occupation showed to be significantly correlated with health literacy. Therefore, promoting pregnant women’s health literacy through simplifying health-related information, use of audio-visual media, improving communicative skills among the health staff and mothers can be effective.

## Introduction

Health literacy deals with one’s capacity of achieving, processing and understanding the fundamental information and services required for making proper health-related decisions (1). According to WHO, health literacy has to do with socio-cognitive skills which determine one’s motivation for and capability of achieving, understanding and employing the information needed for promoting the healthcare (2). Global works of research attest to the fact that through improving such factors as the level of education and health literacy, social services and economy, both individual and social health levels can be positively influenced (3). The twentieth century is marked by a special attention to health literacy as a global issue. In a recent report, WHO introduced health literacy as one of the greatest determinants of healthcare (4). Health literacy is of an utmost importance in different target groups including the elderly and adults. One significant yet vulnerable social group concerning this is pregnant women. Health literacy is considered as a key element enabling women to engage in self-care as well as child-care activities. Without adequate knowledge about healthcare, informed decisions with desirable consequences are hard to make (5). A woman’s state of health and healthcare information or in other words, her health literacy before, during and after pregnancy over years directly affect her child. Educating women is essential to improve the health state of their children and family. That is why women are known as the primary population to promote health literacy (6). An investigation revealed that patients with inadequate health literacy are less capable of understanding medical information leaflets, indications of a drug and so on (7). Some other research indicated the direct effect of mothers’ health literacy on the fetus (1). Still another study showed the positive correlation of low health literacy and unintended pregnancy (8). Shieh et al. observed that low health literacy negatively influences women’s contraceptive behavior, healthcare activities and childcare (5). A body of research reported on the low state of health literacy in Iranian women (9-11). Due to the high significance of health literacy during pregnancy and its direct effect on the fetus and the limited research addressing this issue in Iran, the present research aimed to investigate the health literacy of pregnant women in the South of Iran.

## Materials

The research population of the present descriptive-analytical study was all pregnant women in four southern provinces of Iran. A body of previous research reported a 45% of adequate health literacy. With a confidence level of 95% and an acceptable difference of 5% in the actually adequate health literacy, sample size can be estimated through the formulation bellow:

$n=\frac{z^{2}\mathrm{pq}}{d^{2}}=\frac{{\left(1.96\right)}^{2}\times 0.45\times 0.55}{{\left(0.05\right)}^{2}}\approx 381$

Since the sampling method was clustering, the final sample size was set to be 775 after consulting a statistician. In the clustering, the number of subjects in each cluster was selected to be 25. Thus, 31 clusters were selected overall. Generally speaking, there were 172 healthcare centers (101 in Ahvaz, 37 in Zahedan, 13 Boushehr and 22 in Bandar Abbas) in the four provinces. According to the number of clusters involved and the overall number of healthcare centers, the number of subjects per cluster was determined: Ahvaz (17 clusters, 425 subjects), Zahedan (7 clusters, 175 subjects), Bandar Abbas (4 clusters, 100 subjects) and Boushehr (3 clusters, 75 subjects). To select the subjects in each cluster, simple randomized sampling was followed. The file numbers of all households having a pregnant mother were enlisted and from among them all, 25 files in each cluster were selected randomly. The inclusion criteria were being literate, sharing Iranian nationality, having a medical file for pregnancy care, having no background diseases and no threat to pregnancy. Face-to-face interviews were made with the subjects. To measure their health literacy, the Persian translated version of the Test of Functional Health Literacy in Adults was employed which is a valid instrument for measuring health literacy. The reliability and validity of the test were established for the Iranian population by Tehrani et al. Once translated, the questionnaire was submitted to 50 subjects, and a number of changes were made to some items according to the difficulty level of some items and the variance of responses (12). This questionnaire is comprised of three sections the first of which contains demographic information: age, education, occupation, spouse’s occupation, accommodation, residential area, monthly income, number of previous pregnancies, number of children, time of beginning and continuation of healthcare during pregnancy and the key source of gaining health-related information. The second section deals with reading comprehension and measures the respondent’s ability to read actual texts on healthcare provision. The texts designed for reading comprehension have 50 multiple-choice questions. A correct answer would receive 1 while an incorrect one would receive 0. One’s score from this section of the questionnaire would then range between 0 and 50. 20 minutes were allocated to answer this section. The next section is calculations (mathematical comprehension) which measures one’s capability of dealing with calculating situations. In this section, 10 cards along with notes on prescribed drugs, time of the visit, stages of receiving financial support and an instance of a blood sugar test are presented to the subject and are expected to be responded within 10 minutes. The score of these 17 items was raised to 50 with an application of a coefficient (2.941). The overall score of this questionnaire was to be estimated out of 100. According to the cut-off points, there were three levels of interpreting health literacy: inadequate (0-59), borderline (59-74) and adequate (74-100). Ethical approval was obtained from Ethical Board Committee of shahid beheshti University of Medical Sciences, ethical number SBMS.REC.1395.312. The procedures began with coordination with the security of the target university and was followed by revealing the purpose of research and acquiring subjects’ full consent to participate. Ethical issues were taken into consideration and the subjects were ensured of the confidentiality of the data they produced. They received the questionnaires and responded to the items within the time limit. The data were later analyzed through SPSS ver.16.0. Descriptive statistics (relative and absolute frequency, mean and standard deviation) were used to describe the data. Inferential statistics were also used including a test of correlation, independent-sample t-test and ANOVA. The significance level was set at p ˂ 0.05 in this study.

## Results

The average age of the pregnant women was 31.78 ± 7.61 years and their age ranged between 14 and 58. 36.4% of the subjects had a university degree (Table 1).

The results revealed that the mean score of reading comprehension was 38.64 ± 6.50; the mean score of mathematical calculation comprehension turned out to be 33.83 ± 8.77; the mean score of health literacy showed to be 72.48 ± 14.43. Accordingly, 15.5% of the subjects had an inadequate level of health literacy; 41.7% had a border-line level of health literacy, and 42.8% enjoyed an adequate level of literacy. Significant correlations were observed between mothers’ age, occupation and quality of healthcare and their level of health literacy (p ˂ 0.05) (Tables 2, 3).

**Table 1:** Research subjects’ demographic information

**Variable**	**Group**	**Frequency**	**%**
Occupation	Unemployed (homemaker)	476	61.4
	Employed	299	38.6
Residential area	Rural	214	27.6
	Urban	561	72.4
Accommodation	Own a house	476	61.4
	Rent a house	191	24.6
	Corporate house	48	6.2
	Other	60	7.7
Quality of care	Regular	663	85.5
	Occasional	112	14.5
Source of information	Health staff	552	71.2
	Kith and kin	145	18.7
	The press or mass media	63	8.2
	Other	15	1.9
Education	Elementary/junior high school	113	14.6
	Diploma	380	49
	University degree	282	36.4

**Table 2:** Comparison of the mean health literacy scores and demographic features

**Variable**	**Sub-variable**	**f.**	**Functional health literacy**			**Health literacy level**						**Significance level**
			**Reading ** **comprehension**	**Mathematical ** **comprehension**	**Total score**	**inadequate**		**Border-line**		**Adequate**		
			**Mean ± Std**	**Mean ± Std**	**Mean± Std**	**f**	**%**	**f**	**%**	**f**	**%**	
Age	˂ 20	19	40.52 ± 2.75	35.54 ± 4.38	76.07 ± 6.96	0	0	12	63.2	7	36.8	< 0.001
	21-40	670	38.57 ± 6.72	33.80 ± 8.98	72.37 ± 14.79	112	16.7	263	39.3	295	44	
	41-50	64	40.03 ± 4.89	36.42 ± 5.31	76.45 ± 10.01	0	0	34	53.1	30	46.9	
	˃50	22	35.18 ± 4.08	25.98 ± 8.73	61.17 ± 12.77	8	36.4	14	63.6	0	0	
Education	Elementary/junior high school	113	33.33 ± 7.67	28.99 ± 9.29	62.33 ± 16.46	39	34.5	48	42.5	26	23	< 0.001
	diploma	380	38.26 ± 6.35	33.43 ± 8.93	71.70 ± 14.22	63	16.6	172	45.3	145	38.2	
	University degree	282	41.29 ± 4.46	36.31 ± 7.35	77.61 ± 11.14	18	6.4	103	36.5	161	57.1	
Occupation	Unemployed (homemaker)	476	37.80 ± 6.72	32.84 ± 9.49	70.65 ± 15.33	85	17.9	215	45.2	176	37	< 0.001
	employed	299	39.98 ± 5.88	35.41 ± 7.23	75.40 ± 12.33	35	11.7	108	36.1	156	52.2	
Quality of care during pregnancy	Regular	663	38.52 ± 6.66	33.95 ± 8.37	72.47 ± 14.53	105	15.8	278	41.9	280	42.2	< 0.001
	Occasional	112	39.73 ± 5.42	33.18 ± 9.01	72.55 ± 13.86	15	13.4	45	40.2	52	46.4	
Income (tomans)	˂ 500	109	32.50 ± 8.13	28.60 ± 7.76	61.10 ± 14.96	37	33.9	59	54.1	13	11.9	< 0.001
	500-1 million	230	39.09 ± 5.86	35.12 ± 7.35	74.21 ± 12.82	27	11.7	104	45.2	99	43	
	˃ 1 million	424	39.87 ± 5.45	34.52 ± 9.31	74.39 ± 13.92	56	13.2	154	36.3	214	50.5	

**Table 3:** Comparison of the mean scores of health literacy across the target provinces

**Capital city of the province**	**f.**	**Functional health literacy**			**Health literacy level**					
		**Reading ** **comprehension**	**Mathematical ** **comprehension**	**Total score**	**Inadequate**		**Borderline**		**Adequate**	
		**Mean ± SD**	**Mean ± SD**	**Mean ± Sd**	**f**	**%**	**f**	**%**	**f**	**%**
Ahvaz	425	39.33 ± 6.24	34.63 ± 8.45	73.96 ± 13.97	55	13	167	39.4	202	47.6
Boushehr	75	36.54 ± 7.13	30.81 ± 10.77	67.36 ± 16.43	23	30.7	30	40	22	29.3
Bandar Abbas	100	38.34 ± 7.34	34.56 ± 7.86	72.9 ± 14.85	13	13	42	42	45	45
Zahedan	175	38.09 ± 6.09	32.81 ± 8.78	70.91 ± 13.83	29	16.6	83	47.4	63	36

**Table 4:** Comparison of the average health literacy of pregnant women in terms of some variables (Tukey post hoc test)

**Variable**	**Comparison groups**	**Mean ** **Difference**	**Std. Error**	**Sig.**	**95% Confidence Interval**	
					**Lower Bound**	**Upper Bound**
Elementary	Junior	-23.16334^*^	6.12294	< 0.001	-39.0023	-7.3244
	Diploma	-37.98027^*^	5.69137	< 0.001	-52.7028	-23.2577
	University degree	-37.32760^*^	5.67573	< 0.001	-52.0097	-22.6455
Junior	Diploma	-14.81693^*^	3.14602	< 0.001	-22.9551	-6.6787
	University degree	-14.16426^*^	3.11765	< 0.001	-22.2291	-6.0995
Diploma	University degree	/65267	2.14966	0.990	-4/9081	6.2135

The results of post hoc test showed that there is a significant difference between the mean of health literacy in elementary students with those who have the Junior School, diploma and university degree (p < 0.05). But this difference was not significant between those with graduate and university education (p = 0.99) (Table 4).

Logistic regression analysis was performed for predictive variables in women's health literacy, and variables such as education, age, occupation and place of residence were included in the study. The most effective factor in predicting health literacy, education (OR = 3.445) and the lowest impact of job variables (OR = 0.542) (Table 5).

## Discussion

The present study sought to investigate the health literacy of pregnant women visiting the healthcare centers of the southern provinces of Iran. The results indicated that the highest level of health literacy belonged to Ahvaz and the lowest0 level belonged to Boushehr. Ethnicity and local culture can affect one’s healthcare and health literacy. Familial, social and cultural effects play a vital role in forming one’s attitudes and beliefs and their interaction with the health system. The difference among the target provinces can be due to any of the above-mentioned reasons (13). 

The findings revealed that more than half of the subjects had an inadequate and border-line health literacy. In a body of related research, the target research populations showed to suffer from the same levels of literacy (9, 11, 12, 14-18). This can be a function of differing data collection method, social/cultural features, age, education and the quality of healthcare services. A body of related literature overseas also indicate a wide range of inadequate health literacy in many research populations. As an instance, in a systematic research conducted by Orlow et al. in North America on 85 subjects, about 26% of them showed to have an inadequate level of health literacy while 20% had a health literacy on the border-line (19). Wagner et al. reported the level of health literacy inadequate or border-line among 11.4% of American adults (13). Williams et al.’s research addressed the elderly population afflicted with diabetes and hypertension in two American hospitals. The rate of inadequate health literacy in these hospitals were found to be 44 and 49% respectively for diabetic patients and those suffering from hypertension (20). 

The present findings revealed a significant correlation between mothers’ education and their health literacy which is similar to the research finding reported by Tol et al. (21), Amiresmaili et al. (22), Javadzade et al. (15) and a similar body of research (7, 8, 23-25). A higher level of health literacy among those with a higher education in general shows how one’s education influences the level of health literacy. In fact, general education can be taken as a basis for health literacy. A similar finding has been also reported in other investigations (9, 12, 26-31).

**Table 5:** Prognostic factors of health literacy of pregnant women

**Variable**	**Exp(B)**	**Beta**	**Sig.**	**95% Confidence Interval**	
				**Lower Bound**	**Lower Bound**
Education	7.345	0.338	< 0.001	4/396	10.294
Age	-6.555	-0.161	0.007	-11/309	-1.802
Occupation	2.542	0.071	0.295	-2/224	7.307
Place of residence	3.451	0.088	0.161	-1/380	8.282

As systematic review of health literacy by the agency of research and quality of healthcare services indicated that low health literacy was a severe challenge in the U.S. and is more prevalent among those of lower education (˂ diploma). According to this report, education level is a strong predictor of health literacy (7). As the results of the present study showed, less than half of the academic subjects had an inadequate and border-line health literacy. This indicates that education level cannot be a stable determinant of high health literacy. In some other research, Carthery-Goulart pinpointed that education level cannot be a single precise predictor of reading comprehension (23). The present finding was consistent with that of Ghanbari et al. (9) and a body of related research (5, 8). 

A significant negative correlation was found between age and health literacy. In other words, older subjects had lower health literacy. A similar finding has been reported by Michelle et al. (19), Tol (21), Javadzade et al. (15) and others (23-25, 32, 33). The underlying reasons can be: reduced cognitive function, distance from school days, inability of attending the healthcare center regularly and staying updated with the news, loss of physical power, increased physical/social/psychological changes due to aging (9). Moreover, lower health literacy among older women can be related to lower education level. This finding was consistent with Artinian’s (34) and Raisi’s (26). Counterevidence was reported by Ghanbari et al. (9), Peiman and Abdollahi (11) who observed higher health literacy among older subjects. McLaughlin et al. (34) and Anders et al. (8) found no significant correlation between women’s age and health literacy. This can be due to the differing age range in this research and the present research. 

Another finding of the present research was a significant correlation between subjects’ occupation and health literacy. A similar correlation was reported by Tehrani et al. (12). It appears that one’s employment which logically implies a better socio-economic status, is accompanied by higher health literacy. A dissimilar finding was reported by Ghanbari et al. (9) and Peiman and Abdollahi (11). The U.S. national research of health literacy revealed that those functionally illiterate are more probably impoverished, unemployed or holding temporary jobs (33). In the present study, the health staff enjoyed the highest percentage of receiving health-related news. La vonne and Zun observed that the majority of subjects had acquired information from doctors or the healthcare centers (24). To make appropriate health-related decisions, individuals are supposed to comprehend and use the information in exclusively health-related contexts. Healthcare service providers need to be aware of customers’ information processing capabilities. They also need to convey information at different levels matched with patients’ differing levels of health literacy. The present study was an extensive pioneering research in the south of Iran. The other body of research has been conducted on research populations other than pregnant women. Contrary to many other investigations, the present research used the full version of the questionnaire rather than the shortened form, so as to increase the precision and quality of responses. One limitation of this research is concerned with the common problems associated with questionnaire-based data collection. The questionnaire employed was more focused on reading/writing and calculation, while these capabilities are just the basics of health literacy. There are also such other capabilities involved as speech, listening, background knowledge and asking for support for the health system.

## Conclusion

The findings of the present research indicated an inadequate or border-line health literacy in the target population. This can be a warning for mother/child healthcare in Iran since health literacy is a key factor in promoting social health care. There is a crucial need for raising mothers’ health literacy by an initial measurement of their literacy in pregnancy care units, simplifying materials, offering audio-visual instructions to pregnant women and offering textual instructions as posters, pamphlets or brochures. The present research also recommends that the communicative skills of the health staff be promoted. It is hoped that findings of the present pave the way for future research and health literacy be incorporated in detailed or grand national plans within the health system of the country.
